# Intensive short-term dynamic psychotherapy for irritable bowel syndrome: a randomized controlled trial examining improvements in emotion regulation, defense mechanisms, quality of life, and IBS symptoms

**DOI:** 10.3389/fpsyg.2024.1293150

**Published:** 2024-03-28

**Authors:** Faezeh Shafiei, Mahmood Dehghani, Fahimeh Fathali Lavasani, Mehdi Manouchehri, Marjan Mokhtare

**Affiliations:** ^1^Department of Clinical Psychology, School of Behavioral Science and Mental Health (Tehran Institute of Psychiatry), Iran University of Medical Sciences, Tehran, Iran; ^2^Department of Psychology, Faculty of Medical, Tehran Medical Sciences, Islamic Azad University, Tehran, Iran; ^3^Gastroenterology and Hepatology, Internal Medicine Department, Iran University of Medical Sciences, Tehran, Iran

**Keywords:** IBS, ISTDP, psychodynamic psychotherapy, emotion regulation, defense mechanisms, quality of life

## Abstract

Studies have provided evidence for the effectiveness of intensive short-term dynamic psychotherapy (ISTDP) in treating medically unexplained symptoms (MUS). This study aimed to examine the effectiveness of ISTDP on individuals living with irritable bowel syndrome (IBS) in terms of, emotion regulation (ER) abilities, defense mechanisms, quality of life (QOL), and IBS symptoms. A total of 30 patients diagnosed with IBS were recruited and randomly assigned to either the intervention (*n* = 15) or control (*n* = 15) group. Pre- and post-treatment assessments were conducted, along with a follow-up assessment after ten weeks. Repeated measures analyses of variance were employed to analyze the data. The findings revealed that ISTDP led to significant improvements in ER, defense mechanisms, and QOL, as well as a reduction in the severity and frequency of IBS symptoms. These results provide further support for the efficacy of ISTDP as a treatment modality for individuals with IBS.

## Introduction

Irritable bowel syndrome (IBS) is a chronic functional gastrointestinal disorder (FGD) that involves recurring instances of abdominal pain, discomfort, and changes in bowel movements that are not explained by any biochemical or structural irregularities (Chey et al., 2015). IBS is currently one of the most common medical disorders encountered by healthcare providers, affecting up to 20% of the population in developed countries (Chang et al., 2010; Bokic et al., 2015). Within primary care settings, around 12% of individuals seek medical attention for IBS-related issues, establishing it as the predominant subgroup observed in gastroenterology clinics (Saha, 2014). While IBS is not life-threatening, it can significantly impair a person’s life and hinder daily functioning, leading to high rates of work absenteeism, hospital visits, and physician appointments. Additionally, severe cases of IBS pose a substantial financial burden on individuals. This strain comes from recurring costs for consultations, tests, medications, and additional treatments. Lost income due to reduced work productivity and absenteeism, plus expenses for mental health support and specialized care, also contribute. In summary, managing severe IBS affects physical and mental well-being, and creates considerable financial challenges (Jung et al., 2014; Chey et al., 2015).

The exact cause of IBS remains elusive, posing a challenge for physicians in identifying suitable treatment options. Thus far, many treatments have proven ineffective, highlighting the complexity of addressing this condition. However, recent research indicates that the pathogenesis of IBS involves a complex interplay of genetic factors, environmental influences, microbiota composition, and immune response (Loddo and Romano, 2015; Guan, 2019; Eijsbouts et al., 2021). Concurrently, there is emerging evidence suggesting a correlation between the development of IBS and various psychological variables. Individuals with IBS commonly experience psychological disorders, pathological personality traits, and mental health issues (Zomorrodi et al., 2015; Knowles et al., 2017; Pellissier and Bonaz, 2017). A study has shown that between 50 to 90% of IBS patients seeking treatment have a history of mental health disorders, including major depressive disorder, generalized anxiety disorder, panic disorder, social anxiety disorder, somatization disorder, and post-traumatic stress disorder (Keough et al., 2011). Another study found that 25% of IBS patients reported somatization disorder (Craske et al., 2011). Therefore, psychological disturbances may represent a significant risk factor for the development or exacerbation of gastrointestinal symptoms in individuals with IBS.

Research has found a correlation between IBS and emotional processing difficulties, particularly with alexithymia, which refers to difficulty identifying, describing, and experiencing emotions (e.g., Kano et al., 2007; Phillips et al., 2013; Lee et al., 2017; Sibelli et al., 2018; Selvi and Bozo Özen, 2022). These findings suggest that individuals with IBS may experience challenges with regulating their emotions. Emotion regulation (ER) pertains to an individual’s capacity to modulate emotional arousal and engage in goal-directed behaviors regardless of their emotional state. Deficits in ER, or emotion dysregulation (ED), can lead to challenges in monitoring, evaluating, or adapting emotional reactions (Gratz and Roemer, 2004; Gross, 2013). Research has identified adaptive ER as a protective factor against psychological problems, while ED is considered a vulnerability factor (Gross and Muñoz, 1995; Gross and Jazaieri, 2014). Furthermore, although few studies have been conducted on the subject, some have found a positive relationship between ED and gastrointestinal symptoms (Mazaheri, 2015; Sibelli et al., 2018; Selvi and Bozo Özen, 2022). Studies have indicated relationships between ED and susceptibility to physical illnesses such as breast cancer, rheumatoid arthritis, and infertility (Yıldız and Duy, 2019). For instance, research has linked ED and the experience of negative emotions, such as anger and anxiety, to exacerbation of breast cancer (Brandão et al., 2016), rheumatoid arthritis (van Middendorp et al., 2005), and chronic pain (Koechlin et al., 2018). Additionally, some studies have shown that difficulties in conscious experience, regulation, and expression of emotions are associated with the onset and progression of somatoform disorders (Begley, 1994). Moreover, other studies have suggested that anger can significantly impact antral intestinal movement activity in individuals with IBS (Stanculete et al., 2019). In one study, it was found that IBS patients who do not employ anger suppression as a coping mechanism may experience increased abdominal pain and more severe bowel movements following meals (Prat et al., 1996).

Also, the way in which patients with IBS react to emotional events may be influenced by their use of defense mechanisms, which are automatic psychological processes that help individuals cope with stress (American Psychiatric Association, 2013). Limited research has assessed defense mechanisms in patients with IBS, but some studies have found that they tend to use more immature defense mechanisms, such as projection and passive-aggression, and less mature mechanisms compared to healthy individuals (Saeed et al., 2019). Specifically, patients with IBS are more likely to use escape-avoidance and turning-against-self mechanisms, which may indicate that they are consciously trying to avoid or escape problems instead of effectively coping with them. The use of immature defense mechanisms, such as turning-against-self and passive-aggression, may be a form of self-punishment for patients with IBS, which may lead to the exacerbation of IBS symptoms (Ihilevich and Gleser, 1986; Pokroy et al., 1999; Saeed et al., 2019). Furthermore, IBS significantly impacts an individual’s quality of life (QOL) through various mechanisms. Research indicates that IBS can lead to a decreased QOL due to its debilitating symptoms, such as abdominal pain, altered bowel habits, and unpredictability of symptom onset (e.g., Trindade et al., 2022). These symptoms often result in heightened levels of depression, anxiety, and psychological distress, further diminishing QOL (Kopczyńska et al., 2018). IBS can also disrupt daily life, affecting work productivity and social engagements (Kopczyńska et al., 2018; Trindade et al., 2022). All these taken into account, addressing these psychological factors, such as through psychotherapy, may be important in the management and treatment of IBS. By targeting these underlying variables, individuals with IBS may experience improvements in their physical symptoms and overall quality of life.

The limited success of medical treatments for IBS has led to the emergence of various psychological therapies (Guthrie et al., 1993; Hetterich and Stengel, 2020). Meanwhile, multiple systematic reviews and meta-analyses have demonstrated the significant efficacy of psychotherapy in improving IBS symptoms and daily functioning (Laird et al., 2016, 2017; Weibert and Stengel, 2019; Shah et al., 2020). For instance, psychodynamic therapies provide a well-defined framework for addressing the unconscious emotional processing that plays a central role in MUS, and they have been further refined based on neuroscientific findings (Cooper et al., 2017). These therapies specifically focus on emotional and relational processing, aiming to explore the connections between unresolved conflicts and past adverse experiences (Cretton et al., 2020). Several meta-analyses showed that short-term psychodynamic psychotherapies (STPPs) were found to be effective in treating MUS and should be included in treatment guidelines (Lilliengren, 2017; Abbass et al., 2021). Intensive short-term dynamic psychotherapy (ISTDP) is among the most extensively researched STPPs for MUS. A review by Russell et al. (2022) on 11 randomized controlled trials, two control trials, and ten case series studies that examined the use of ISTDP for MUS suggested that ISTDP holds promise as a treatment modality for MUS. Nevertheless, despite evidence for the effectiveness of ISTDP on MUS, there is currently a lack of research examining the effectiveness of this treatment approach for individuals living with IBS specifically. ISTDP theory suggests that somatic symptoms in patients with IBS may result from anxiety caused by the unconscious avoidance of emotional experiences (Abbass et al., 2012). The treatment approach of ISTDP involves helping patients deal with their unconscious conflicts and emotions. Patients with IBS exhibit similar characteristics to those with other MUS conditions, including high levels of negative emotions, emotional dysregulation, and dysfunctional defense mechanisms. As a result, ISTDP may offer a promising treatment option for this group of patients. In an attempt to fill this gap in the literature, this study aimed to examine the effectiveness of ISTDP in improving emotion regulation, defense mechanisms, quality of life, and disease symptoms in individuals with IBS.

## Methods

### Participants and procedure

**Figure 1:**
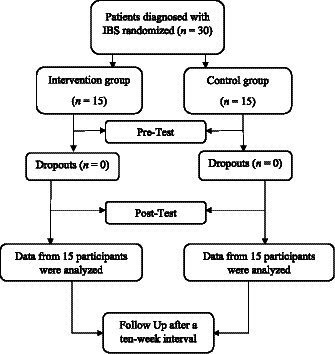
Process chart from recruitment to follow-up measurement.

A randomized pre-test, post-test, and follow-up (RPPF) design with an intervention and a control group was used in this study. Participants were recruited from the gastroenterology clinic of Hazrat Rasool Hospital in Tehran using purposeful sampling methods. A total of 30 patients who met the diagnosis of IBS based on the judgment of gastroenterology specialists using the Rome III criteria were recruited. Participants were randomly assigned to either the intervention (*n* = 15) or control (*n* = 15) group. A graphic depiction of the recruitment process is presented in Figure 1. The inclusion criteria required participants to have a diagnosis of IBS based on the judgment of gastroenterology specialists and Rome III criteria, be between 20 and 50 years old, and have at least a high school diploma. Exclusion criteria were serious physical illness, psychiatric disorders with symptoms of psychosis [assessed by the Structured Clinical Interview for DSM-IV (SCID-l; First and Gibbon, 2004)], and absence of more than two treatment sessions. Both groups received usual medical care, while the control group was informed that they were on a waiting list and would receive treatment after a specified time. Participants provided informed consent after being informed about the aims and procedure of the study, as well as the confidentiality of their data. Participants completed pre-questionnaires and then received 16 weekly individual psychotherapy sessions based on the published treatment manual (Frederickson, 2013). Both groups completed post-questionnaires at the end of the 16th week and then completed questionnaires again after a ten-week interval. This study was first reviewed and approved by the Research Deputy of Iran University of Medical Sciences (Code Number = IR.IUMS.REC.1400.566) and was retrospectively registered in the Iranian Registry of Clinical Trials (ID Number = IRCT20221101056369N1, Registration Date: 2023-07-25).

### Intervention

#### Intensive short-term dynamic psychotherapy

ISTDP is an active therapeutic approach that involves identifying the patient’s defensive system and making them aware of its self-sabotaging consequences in their life. This process helps the patient turn against their defenses and mobilize their own will to overcome pathogenic forces. The therapist confronts the patient respectfully but relentlessly, which leads to the emergence of an unconscious therapeutic alliance. At the same time, this work with defenses mobilizes unresolved feelings in the transference and triggers corresponding anxiety. Davanloo, the founder of ISTDP identified three neurobiological discharge pathways of unconscious anxiety and the process of motor conversion. The first pathway is observable as hand clenching and sighing respirations, accompanied by what he called “isolation of affect” where clients primarily use intellectual awareness devoid of emotional experience. The second pathway affects the muscles of the gastrointestinal tract, blood vessels, and airways, resulting in problems such as migraines, irritable bowel syndrome, and hypertension, and leads to instant repression of emotions and major depression. The third pathway leads to cognitive perceptual disruption, where the person experiences visual blurring, mental confusion, and hallucinations. Clients with motor conversion, with focal or global muscle weakness, also experience repression of emotions. In ISTDP, it is crucial to continuously observe the neurobiological channels of anxiety and the patient’s tolerance capacity to keep the process within their capacity. The process helps the patient experience their repressed feelings in the transference, leading to a subsequent shift to the person in their life toward whom the repressed feelings were generated. Through this process, the patient can work through the corresponding feelings of rage, guilt, grief, and affection, overcome their defensive system, and improve their tolerance capacity. The patient’s will poses a challenge and pressure to overcome defenses and anxiety and to experience their repressed feelings (Frederickson, 2013).

Our intervention incorporated a psychoeducational component, specifically educating participants about the triangle of conflict (i.e., defense, anxiety, and feeling) using insights gleaned from patient communications during the sessions. However, the intervention did not include homework assignments. To ensure consistency and adherence to the study protocol, the ISTDP treatment followed a meticulously manualized approach delivered identically across all participants, regardless of their initial level of distress. Every participant received the full 16 planned sessions, a fact that was communicated before the commencement of therapy. Each session lasted 50 min, with initial sessions extending to 1.5 h per participant. As the manualized intervention predetermined conclusion after 16 sessions, this endpoint was conveyed to participants initially. Thus, no specific indicators were used to determine therapy completion, as it was based solely on the predetermined session count.

### Intervention instructor

The ISTDP sessions were facilitated by the first author, a doctoral candidate in clinical psychology, who had undergone extensive training and continued mentoring in this therapeutic approach. With the full consent of those participating, all treatment sessions were documented through audio recordings to enable examination of procedural integrity and protocol adherence. The supervising faculty member carried out periodic reviews of randomly selected recorded sessions as a means to guarantee the uniformity of the administered interventions. This oversight mechanism enabled ongoing quality assurance and standardization of the supplied therapeutic techniques.

### Outcome measures

#### The irritable bowel syndrome quality of life

The IBS-QOL is a self-report measure of quality of life for patients with IBS (Patrick et al., 1998). It has 34 items rated on a five-point Likert scale ranging from 1 (*“not at all”*) to 5 (*“extremely”*) and load on eight domains: dysphoria, interference with activities, body image, health worry, food avoidance, social reactions, sexual health, and effect on relationships. The Persian version of IBS-QOL showed acceptable psychometric properties (Masaeli et al., 2013).

#### Difficulties in emotion regulation scale

The DERS (Gratz and Roemer, 2004) is a 36-item self-report questionnaire that assesses emotion dysregulation. The DERS items load on six subscales, including Lack of Emotional Awareness (6 items), Lack of Emotional Clarity (5 items), Difficulties Controlling Impulsive Behaviors When Distressed (6 items), Difficulties Engaging in Goal-Directed Behavior When Distressed (5 items), Nonacceptance of Negative Emotional Responses (6 items), and Limited Access to Effective ER Strategies (8 items). Participants rate items on a 5-point scale ranging from 1 (*almost never*) to 5 (*almost always*). A total score is obtained by summing all items. The internal consistency and validity of the Persian version of DERS were supported with the Iranian sample in previous studies (Besharat and Bazzazian, 2013; Vafa et al., 2021).

#### Defensive styles questionnaire

The DSQ-40 (Andrews et al., 1993) is a self-report measure of defense mechanisms, which comprises 40 items rated on a 9-point Likert scale. The 40 items are used to derive scores for 20 defense mechanisms, with two items for each. Immature, neurotic, and mature defensive styles scores are yielded by averaging the ratings for relevant items. The Persian version of the DSQ-40 yielded acceptable psychometric properties (Heidari Nasab et al., 2007).

### Data analyses

We used SPSS 20 software for data entry and statistical analyses. The normality of the distribution for outcome measures was tested using the Kolmogorov–Smirnov test, and the results supported the normality of the data (*p >* 0.05). We then analyzed the pre-test differences in demographic and outcome variables between the two groups via the independent *t*-tests for continuous variables and chi-squared tests for categorical variables. We analyzed the outcome measures by means of repeated measures ANOVAs, with the group as between-subject factor and the time (pre-test, post-test, and follow-up) as within-subject factors. For testing the assumption of the sphericity in repeated-measures ANOVA, we examined the epsilon (ε) value; if the ε value did not fall in the acceptable range (i.e., 0.75 <), we relied on the Huynh-Feldt correction when reporting the results (Girden, 1992). The following rules of thumb are used to interpret values for partial eta squared: *η_p_*^2^ = 0.01 indicates a small effect; *η_p_*^2^ = 0.06 indicates a medium effect; *η_p_*^2^ = 0.14 indicates a large effect. When necessary, post-hoc tests were performed to conduct detailed pairwise comparisons between assessments across the groups, with Bonferroni adjustment applied.

## Results

As shown in Table 1 groups did not differ significantly on demographic variables, including age, gender distribution, and level of education. Thus, the groups were matched in these variables. Table 2 also presents the means and standard deviations of study variables across assessment steps in each group.

**Table 1:** The comparison of demographic data between the intervention and control groups.

	Groups		Comparison	
Variables	Intervention (*n* = 15)	Control (*n* = 15)	*t/Χ*^2^	*p*
Age, Mean (*SD*)	39.67 (6.18)	38.22 (5.48)	0.68	0.502
*Gender (%)*				
Male	7 (46.70)	6 (40.00)		
Female	8 (53.30)	9 (60.00)	0.14	0.713
*Education (%)*				
Diploma	4 (26.70)	5 (33.30)		
Bachelor	6 (40.00)	7 (46.70)	0.169	0.709
Master and above	5 (33.30)	3 (20.00)		

**Table 2:** Descriptive statistics of study variables across pre-test, post-test, and follow-up assessments.

Variable	Group	Pre-test	Post-test	Follow-up
		Mean (*SD*)	Mean (*SD*)	Mean (*SD*)
DERS	Intervention	120.93 (8.55)	103.93 (12.89)	102.40 (11.68)
	Control	122.60 (7.62)	119.73 (9.48)	119.66 (7.81)
Accept	Intervention	20.93 (3.10)	17.60 (20.94)	17.46 (2.94)
	Control	20.73 (3.34)	20.26 (3.08)	20.53 (3.31)
Goal	Intervention	18.73 (2.54)	15.73 (2.21)	15.66 (2.71)
	Control	19.26 (3.28)	18.93 (2.25)	19.20 (2.17)
Impulse	Intervention	20.73 (2.96)	17.60 (4.17)	17.00 (3.27)
	Control	21.13 (2.69)	20.80 (2.45)	20.40 (2.35)
Strategy	Intervention	25.73 (3.55)	24.00 (4.56)	24.46 (4.37)
	Control	21.13 (2.69)	20.80 (2.45)	20.40 (2.35)
Aware	Intervention	18.46 (2.32)	15.53 (3.22)	14.86 (3.99)
	Control	19.26 (2.60)	18.26 (2.68)	18.13 (2.82)
Clarity	Intervention	16.33 (2.60)	13.46 (3.09)	12.93 (2.12)
	Control	16.53 (2.47)	16.13 (2.69)	15.86 (2.41)
Immature	Intervention	122.00 (32.70)	94.46 (26.67)	83.13 (26.23)
	Control	122.86 (29.70)	119.20 (29.13)	118.66 (27.38)
Neurotic	Intervention	48.06 (8.78)	37.86 (9.80)	31.73 (7.39)
	Control	45.86 (8.40)	47.53 (8.82)	46.86 (6.06)
Mature	Intervention	32.86 (10.74)	45.20 (13.73)	52.60 (9.83)
	Control	31.60 (11.34)	33.13 (9.33)	34.60 (8.65)
Quality of life	Intervention	115.73 (8.63)	108.20 (7.58)	106.93 (6.90)
	Control	115.66 (6.74)	111.13 (6.24)	111.60 (5.30)
Social reactions	Intervention	14.20 (2.42)	13.46 (2.74)	13.20 (1.16)
	Control	14.13 (2.41)	14.00 (1.85)	14.06 (1.70)
Interference with activities	Intervention	25.26 (4.13)	23.40 (3.54)	23.46 (3.29)
	Control	24.93 (3.41)	23.13 (2.26)	23.60 (2.69)
Health worry	Intervention	9.86 (2.23)	9.13 (2.06)	8.53 (1.24)
	Control	10.06 (1.66)	9.73 (1.27)	9.46 (1.12)
Body image	Intervention	14.40 (2.41)	13.13 (2.16)	13.06 (2.01)
	Control	14.73 (2.28)	13.86 (1.45)	13.93 (1.33)
Food avoidance	Intervention	6.60 (1.24)	6.00 (1.25)	6.40 (1.12)
	Control	6.73 (1.09)	6.33 (0.97)	6.53 (1.12)
Effect on relationships	Intervention	14.00 (2.75)	13.53 (1.68)	13.06 (1.43)
	Control	14.13 (2.58)	13.93 (1.53)	13.86 (1.59)
Sexual health	Intervention	5.86 (1.24)	5.66 (1.17)	5.73 (1.16)
	Control	6.00 (1.19)	5.86 (1.12)	5.93 (1.38)
Dysphoria	Intervention	25.53 (4.38)	23.86 (3.99)	23.46 (3.75)
	Control	24.93 (4.11)	24.26 (3.26)	24.20 (2.83)
Severity of IBS symptoms	Intervention	11.93 (1.70)	9.86 (1.95)	8.33 (1.58)
	Control	11.80 (1.93)	12.06 (1.33)	11.86 (1.59)
Frequency of IBS symptoms	Intervention	12.46 (2.38)	10.20 (2.21)	9.00 (1.73)
	Control	12.26 (2.01)	12.53 (2.09)	12.66 (2.38)

SD, Standard deviation; DERS, Difficulties in Emotion Regulation Scale; Accept, Non-acceptance of Negative Emotional Responses; Goal, Difficulties Engaging in Goal-Directed Behavior; Impulse, Difficulties Controlling Impulsive Behaviors; Strategies, Limited Access to Emotion Regulation strategies; Aware, Lack of emotional awareness; Clarity, lack of emotional clarity; IBS, Irritable bowel syndrome.

Repeated measures ANOVAs were conducted to examine the effectiveness of ISTDP on emotion regulation difficulties. As shown in Table 3, the results demonstrated a significant main effect of time for the DERS total score [*F* (2, 56) = 24.68, *p* < 0.001, *η_p_*^2^ = 0.47] and subscales of Nonacceptance of Negative Emotional Responses [*F* (2, 56) = 4.79, *p* < 0.01, *η_p_*^2^ = 0.15], Difficulties Engaging in Goal-Directed Behavior When Distressed [*F* (2, 56) = 4.81, *p* < 0.02, *η_p_*^2^ = 0.15], Difficulties Controlling Impulsive Behaviors When Distressed [*F* (2, 56) = 10.68, *p* < 0.001, *η_p_*^2^ = 0.28], Lack of Emotional Awareness [*F* (2, 56) = 12.89, *p* < 0.001, *η_p_*^2^ = 0.31], and Lack of Emotional Clarity [*F* (2, 56) = 8.27, *p* < 0.001, *η_p_*^2^ = 0.23] indicating that there are significant differences between the assessment steps in these scores. The results also showed significant between-subject effect of group on the DERS total score [*F* (1, 28) = 14.66, *p* < 0.001, *η_p_*^2^ = 0.34] and subscales of Nonacceptance of Negative Emotional Responses [*F* (1, 28) = 4.74, *p* < 0.04, *η_p_*^2^ = 0.15], Difficulties Engaging in Goal-Directed Behavior When Distressed [*F* (1, 28) = 14.99, *p* < 0.001, *η_p_*^2^ = 0.35], Difficulties Controlling Impulsive Behaviors When Distressed [*F* (1, 28) = 6.08, *p* < 0.02, *η_p_*^2^ = 0.18], Lack of Emotional Awareness [*F* (1, 28) = 5.95, *p* < 0.02, *η_p_*^2^ = 0.17], and Lack of Emotional Clarity [*F* (1, 28) = 7.23, *p* < 0.01, *η_p_*^2^ = 0.20], indicating significant overall differences between groups in these scores (Table 3). In addition, there was a significant time × group interaction for the DERS total score [*F* (2, 56) = 12.80, *p* < 0.001, *η_p_*^2^ = 0.34] and subscales of Nonacceptance of Negative Emotional Responses [*F* (2, 56) = 3.27, *p* < 0.04, *η_p_*^2^ = 0.10], Difficulties Engaging in Goal-Directed Behavior When Distressed [*F* (2, 56) = 3.72, *p* < 0.04, *η_p_*^2^ = 0.12], Difficulties Controlling Impulsive Behaviors When Distressed [*F* (2, 56) = 5.47, *p* < 0.007, *η_p_*^2^ = 0.16], Lack of Emotional Awareness [*F* (2, 56) = 3.38, *p* < 0.04, *η_p_*^2^ = 0.11], and Lack of Emotional Clarity [*F* (2, 56) = 4.05, *p* < 0.02, *η_p_*^2^ = 0.13], showing that the changes in the dependent variables across the assessment steps were statistically different between the groups. Post-hoc Tukey test comparisons were performed across the three assessment scores and separately for each group with Bonferroni’s correction for multiple comparisons. As shown in Table 4, our results indicated significant differences from pre-test to post-test and pre-test to follow-up in DERS total and subscales scores of Nonacceptance of Negative Emotional Responses, Difficulties Engaging in Goal-Directed Behavior When Distressed, Difficulties Controlling Impulsive Behaviors When Distressed, Lack of Emotional Awareness, and Lack of Emotional Clarity (*p* < 0.001 to 0.037) only in the intervention group (Table 4).

**Table 3:** The results of repeated measures ANOVAs.

Dependent Variable	Source	Type III Sum of Squares	df	Mean Square	F	*p*	*η_p_^2^*
DERS	Time	2145.156	2	1072.578	24.680	<0.001	0.468
	Group	3016.011	1	3016.011	14.667	0.001	0.344
	Time × Group	1113.156	2	556.578	12.807	<0.001	0.314
Accept	Time	69.75	2	34.878	4.788	0.012	0.146
	Group	76.544	1	76.544	4.743	0.038	0.145
	Time × Group	47.622	2	23.811	3.269	0.045	0.105
Goal	Time	52.422	1.502	34.901	4.812	0.021	0.147
	Group	132.011	1	132.011	14.999	0.001	0.349
	Time × Group	40.556	1.502	27.000	3.723	0.044	0.117
Impulse	Time	82.422	2	41.211	10.682	<0.001	0.276
	Group	122.500	1	122.500	6.079	0.020	0.178
	Time × Group	42.200	2	21.100	5.469	0.007	0.163
Strategy	Time	16.689	1.583	10.543	1.112	0.326	0.038
	Group	13.611	1	13.611	0.437	0.514	0.015
	Time × Group	8.289	1.583	5.237	0.552	0.539	0.019
Aware	Time	96.289	2	48.144	12.893	<0.001	0.315
	Group	115.600	1	115.600	5.953	0.021	0.175
	Time × Group	25.267	2	12.633	3.383	0.041	0.108
Clarity	Time	69.622	2	34.811	8.273	0.001	0.228
	Group	84.100	1	84.100	7.233	0.012	0.205
	Time × Group	34.067	2	17.033	4.048	0.023	0.126
Immature	Time	7422.489	2	3711.244	10.900	<0.001	0.280
	Group	9343.211	1	9343.211	4.576	0.041	0.140
	Time × Group	4720.089	2	2360.044	15.055	<0.001	0.350
Neurotic	Time	1969.267	2	984.633	8.254	0.001	0.228
	Group	1276.900	1	1276.900	17.188	0.000	0.380
	Time × Group	1079.622	2	539.811	6.688	0.002	0.193
Mature	Time	885.422	2	442.711	8.897	<0.001	0.241
	Group	2454.444	1	2454.444	11.419	0.002	0.290
	Time × Group	1177.867	2	588.933	17.140	<0.001	0.380
Quality of life	Time	779.489	2	389.744	13.552	<0.001	0.326
	Group	141.878	1	141.878	1.602	0.216	0.054
	Time × Group	86.022	2	43.011	1.496	0.233	0.051
Social reactions	Time	4.822	2	2.411	0.751	0.477	0.026
	Group	4.444	1	4.444	0.578	0.454	0.020
	Time × Group	3.356	2	1.678	0.522	0.596	0.018
Interference with activities	Time	58.867	2	29.433	3.592	0.034	0.114
	Group	4.444	1	4.444	0.578	0.454	0.020
	Time × Group	.956	2	0.478	0.058	0.943	0.002
Health worry	Time	14.067	2	7.033	3.605	0.034	0.114
	Group	7.511	1	7.511	1.730	0.199	0.058
	Time × Group	2.022	2	1.011	0.518	0.598	0.018
Body image	Time	22.756	2	11.378	3.761	0.029	0.118
	Group	9.344	1	9.344	1.611	0.215	0.054
	Time × Group	1.156	2	0.578	0.191	0.827	0.007
Food avoidance	Time	3.800	2	1.900	0.581	0.215	0.053
	Group	0.900	1	0.900	0.602	0.444	0.021
	Time × Group	0.200	2	0.100	0.083	0.920	0.003
Effect on relationships	Time	5.422	2	2.711	0.641	0.531	0.022
	Group	4.444	1	4.444	1.242	0.275	0.042
	Time × Group	1.689	2	0.844	0.200	0.820	0.007
Sexual health	Time	0.422	2	0.211	0.119	0.888	0.004
	Group	0.711	1	0.711	0.793	0.381	0.028
	Time × Group	0.022	2	0.011	0.006	0.994	0.000
Dysphoria	Time	33.756	2	16.878	2.007	0.144	0.067
	Group	0.711	1	0.711	0.028	0.869	0.001
	Time × Group	7.222	2	3.611	0.429	0.653	0.015
Severity of IBS symptoms	Time	46.822	2	23.411	7.921	0.001	0.221
	Group	78.400	1	78.400	28.305	0.000	0.503
	Time × Group	51.667	2	25.833	8.741	<0.001	0.238
Frequency of IBS symptoms	Time	36.356	1.549	23.468	6.620	0.006	0.191
	Group	84.100	1	84.100	10.048	0.004	0.264
	Time × Group	57.867	1.549	37.354	10.536	0.001	0.273

DERS, Difficulties in Emotion Regulation Scale; Accept, Non-acceptance of Negative Emotional Responses; Goal, Difficulties Engaging in Goal-Directed Behavior; Impulse, Difficulties Controlling Impulsive Behaviors; Strategies, Limited Access to Emotion Regulation strategies; Aware, Lack of emotional awareness; Clarity, lack of emotional clarity; IBS, Irritable bowel syndrome.

**Table 4:** *Post hoc* tukey tests to compare three assessment scores across groups.

			Intervention		Control	
Dependent Variable	Time (I)	Time (J)	Mean Difference (I-J)	*p*	Mean Difference (I-J)	*p*
DERS	Pre-Test	Post-Test	17.000	<0.001	2.867	0.688
	Post-Test	Follow-up	18.533	<0.001	2.933	0.623
		Follow-up	1.533	1.00	0.067	1.000
Accept	Pre-Test	Post-Test	3.333	0.012	0.467	1.000
	Post-Test	Follow-up	3.467	0.018	0.200	1.000
		Follow-up	0.133	1.000	−0.267	1.000
Goal	Pre-Test	Post-Test	3.000	0.029	0.333	1.000
	Post-Test	Follow-up	3.067	0.037	0.067	1.000
		Follow-up	0.067	1.000	−0.267	1.000
Impulse	Pre-Test	Post-Test	3.133	0.010	0.333	1.000
	Post-Test	Follow-up	3.733	0.002	0.733	0.683
		Follow-up	0.600	1.000	0.400	1.000
Strategy	Pre-Test	Post-Test	1.733	0.473	0.333	1.000
	Post-Test	Follow-up	1.267	1.000	0.133	1.000
		Follow-up	−0.467	1.000	−0.200	1.000
Aware	Pre-Test	Post-Test	2.933	0.001	1.000	0.642
	Post-Test	Follow-up	3.600	0.001	1.133	0.505
		Follow-up	0.667	0.871	0.133	1.000
Clarity	Pre-Test	Post-Test	2.867	0.003	0.400	1.000
	Post-Test	Follow-up	3.400	0.002	0.667	1.000
		Follow-up	0.533	1.000	0.267	1.000
Immature	Pre-Test	Post-Test	27.533	0.001	3.667	1.000
	Post-Test	Follow-up	38.867	<0.001	4.200	1.000
		Follow-up	11.333	0.109	0.533	1.000
Neurotic	Pre-Test	Post-Test	−12.333	0.032	−1.533	1.000
	Post-Test	Follow-up	−19.733	<0.001	−3.000	0.825
		Follow-up	−7.400	0.076	−1.467	1.000
Mature	Pre-Test	Post-Test	10.200	0.019	−1.667	1.000
	Post-Test	Follow-up	16.333	<0.001	−1.000	1.000
		Follow-up	6.133	0.060	0.667	1.000
Quality of life	Pre-Test	Post-Test	7.533	0.006	4.533	0.098
	Post-Test	Follow-up	8.800	0.012	4.067	0.078
		Follow-up	1.267	1.000	−0.467	1.000
Social reactions	Pre-Test	Post-Test	0.733	0.852	0.133	1.000
	Post-Test	Follow-up	1.000	0.396	0.067	1.000
		Follow-up	0.267	1.000	−0.067	1.000
Interference with activities	Pre-Test	Post-Test	1.867	0.335	1.800	0.397
	Post-Test	Follow-up	1.800	0.385	1.333	0.820
		Follow-up	−0.067	1.000	−0.467	1.000
Health worry	Pre-Test	Post-Test	0.733	0.625	0.333	1.000
	Post-Test	Follow-up	1.333	0.101	0.600	0.984
		Follow-up	0.600	0.597	0.267	1.000
Body image	Pre-Test	Post-Test	1.267	0.368	0.867	0.529
	Post-Test	Follow-up	1.333	0.372	0.800	0.395
		Follow-up	0.067	1.000	−0.067	1.000
Food avoidance	Pre-Test	Post-Test	0.600	0.685	0.400	0.865
	Post-Test	Follow-up	0.200	1.000	0.200	1.000
		Follow-up	−0.400	1.000	−0.200	1.000
Effect on relationships	Pre-Test	Post-Test	0.467	1.000	0.200	1.000
	Post-Test	Follow-up	0.933	0.947	0.267	1.000
		Follow-up	0.467	1.000	0.067	1.000
Sexual health	Pre-Test	Post-Test	0.200	1.000	0.133	1.000
	Post-Test	Follow-up	0.133	1.000	0.067	1.000
		Follow-up	−0.067	1.000	−0.067	1.000
Dysphoria	Pre-Test	Post-Test	1.667	0.239	0.667	1.000
	Post-Test	Follow-up	2.067	0.397	0.733	1.000
		Follow-up	0.400	1.000	0.067	1.000
Severity of IBS symptoms	Pre-Test	Post-Test	2.067	0.009	−0.267	1.000
	Post-Test	Follow-up	3.600	<0.001	−0.067	1.000
		Follow-up	1.533	0.064	0.200	1.000
Frequency of IBS symptoms	Pre-Test	Post-Test	2.267	0.021	−0.267	1.000
	Post-Test	Follow-up	3.467	0.001	−0.400	1.000
		Follow-up	1.200	0.017	−0.133	1.000

DERS, Difficulties in Emotion Regulation Scale; Accept, Non-acceptance of Negative Emotional Responses; Goal, Difficulties Engaging in Goal-Directed Behavior; Impulse, Difficulties Controlling Impulsive Behaviors; Strategies, Limited Access to Emotion Regulation strategies; Aware, Lack of emotional awareness; Clarity, lack of emotional clarity; IBS, Irritable bowel syndrome.

We conducted three sets of repeated measures ANOVAs to examine the effectiveness of ISTDP on defense mechanisms, and the results showed a significant main effect of time for the immature [*F* (2, 56) = 10.90, *p* < 0.001, *η_p_*^2^ = 0.28], neurotic [*F* (2, 56) = 8.25, *p* < 0.01, *η_p_*^2^ = 0.23], and mature [*F* (2, 56) = 8.90, *p* < 0.001, *η_p_*^2^ = 0.24] defenses. Also, as shown in table, results indicated the significant between-subject effect of group on the immature [*F* (1, 28) = 4.58, *p* < 0.04, *η_p_*^2^ = 0.14], neurotic [*F* (1, 28) = 17.19, *p* < 0.001, *η_p_*^2^ = 0.38], and mature [*F* (1, 28) = 11.42, *p* < 0.002, *η_p_*^2^ = 0.29] defenses. The results were also indicative of significant time × treatment interaction for the immature [*F* (2, 56) = 15.05, *p* < 0.001, *η_p_*^2^ = 0.35], neurotic [*F* (2, 56) = 6.69, *p* < 0.002, *η_p_*^2^ = 0.19], and mature [*F* (2, 56) = 17.14, *p* < 0.001, *η_p_*^2^ = 0.38] defenses (Table 3). In addition, as could be retrieved from Table 4, post-hoc Tukey test comparisons showed significant differences from pre-test to post-test and pre-test to follow-up in immature, neurotic, and mature defenses (*p* < 0.001 to 0.032) in the intervention group.

We also conducted repeated measures ANOVAs to test the effectiveness of ISTDP on QOL. As Table 3 shows, the results indicated the significant main effect of time for the IBS-QOL total score [*F* (2, 56) = 13.55, *p* < 0.001, *η_p_*^2^ = 0.33] and subscales of interference with activities [*F* (2, 56) = 3.59, *p* < 0.03, *η_p_*^2^ = 0.11], health worry [*F* (2, 56) = 3.60, *p* < 0.03, *η_p_*^2^ = 0.11], and body image [*F* (2, 56) = 3.76, *p* < 0.03, *η_p_*^2^ = 0.12]. No significant between-subject effect of group and time × treatment interaction was observed for the IBS-QOL scores. Post-hoc Tukey test comparisons showed significant differences from pre-test to post-test and pre-test to follow up for only the IBS-QOL total score in the intervention group (Table 4).

Finally, two sets of repeated measures ANOVAs were conducted to examine the effectiveness of ISTDP on the severity and frequency of IBS symptoms. Table 3 shows the significant main effect of time on the severity [*F* (2, 56) = 7.92, *p* < 0.001, *η_p_*^2^ = 0.22] and frequency [*F* (2, 56) = 6.62, *p* < 0.006, *η_p_*^2^ = 0.19] of IBS symptoms. Also, as shown in table, results indicated the significant between-subject effect of the group on the severity [*F* (1, 28) = 28.30, *p* < 0.001, *η_p_*^2^ = 0.50], and frequency [*F* (1, 28) = 10.05, *p* < 0.004, *η_p_*^2^ = 0.26] of IBS symptoms. The results were also supportive of significant time × treatment interaction for the severity [*F* (2, 56) = 8.74, *p* < 0.001, *η_p_*^2^ = 0.24] and frequency [*F* (2, 56) = 10.54, *p* < 0.001, *η_p_*^2^ = 0.27] of IBS symptoms. In addition, as could be retrieved from Table 4, post-hoc Tukey test comparisons showed significant differences from pre-test to post-test and pre-test to follow up for the severity and frequency of IBS symptoms (*p* < 0.001 to 0.02) in the intervention group (Table 4).

## Discussion

This study aimed to examine the effectiveness of ISTDP in improving ER, defense mechanisms, quality of life, and IBS symptoms in individuals with IBS. The results indicated that ISTDP was significantly effective in improving emotion regulation, defense mechanisms, and quality of life, and reducing the frequency/severity of IBS symptoms.

Overall, research indicates that ED problems among patients with IBS could exacerbate the severity/frequency of the IBS symptoms (e.g., Kano et al., 2007; Phillips et al., 2013; Lee et al., 2017; Sibelli et al., 2018; Selvi and Bozo Özen, 2022). In this vein, our findings provide evidence that ISTDP is effective in significantly improving ER abilities in individuals with IBS which is in line with theory and prior research on the effectiveness of the ISTDP on ER (e.g., Frederickson et al., 2018; Town et al., 2019; Malda Castillo et al., 2022). ISTDP facilitates the development of a deeper understanding of emotional experiences, triggers, and patterns. By gaining insight into their emotions, individuals enhance their awareness of emotional states, enabling them to recognize and respond to them more effectively. Additionally, ISTDP supports the processing and integration of unresolved emotional experiences and conflicts (Cooper et al., 2017). This therapeutic process encourages individuals to explore and express their emotions in a safe and supportive environment, leading to a reduction in emotional distress and an enhancement in their capacity to regulate emotions (e.g., Town et al., 2019; Malda Castillo et al., 2022). In this vein, a study on the effectiveness of ISTDP for dissociative seizures showed that participants gained increased control over their seizures and experienced fewer and less frequent episodes, partially due to their improved recognition and ability to address triggers. Participants also mentioned acquiring skills and techniques from ISTDP to manage their emotions and bodily sensations, reducing the likelihood of seizures (Malda Castillo et al., 2022). Furthermore, our study revealed that ISTDP led to a significant increase in the utilization of mature defenses, while simultaneously resulting in a significant reduction in the employment of neurotic and immature defenses. In accordance with our findings, Mehboodi et al. (2022) observed a significant increase in mature defense mechanisms and a concurrent decrease in immature defenses among individuals with social anxiety disorder following the application of ISTDP. Theoretically, ISTDP assists individuals in recognizing their defense mechanisms. It enables them to identify and acknowledge the ineffective defenses that are employed in inappropriate contexts, subsequently helping them reduce their reliance on such defenses. Simultaneously, individuals learn to cultivate and utilize mature and appropriate defenses in various situations. Moreover, as individuals in ISTDP engage with their emotions and gain insight into the behaviors and defenses that contribute to the suppression of their feelings, they gradually recognize the diminishing necessity to conceal their emotions using immature defenses (Frederickson et al., 2018).

In addition, our study demonstrated a significant improvement in the QOL of individuals with IBS following the implementation of ISTDP. ISTDP has the potential to assist individuals in effectively managing their IBS symptoms. This leads to a reduction in symptom severity, frequency, and related distress, resulting in an overall enhancement of QOL. ISTDP also aids in the reduction of anxiety, depression, and stress levels commonly experienced by individuals with IBS, thereby contributing to an improved QOL. Moreover, ISTDP facilitates the development of healthier communication patterns, enhances relationship skills, and fosters a more supportive social network. These improved interpersonal dynamics contribute to a higher quality of life by promoting social support and reducing feelings of isolation or alienation (Malda Castillo et al., 2022; Pakdel et al., 2022; Farzadkia et al., 2023). Furthermore, ISTDP was found to positively impact participants’ relationships with both themselves and others (Malda Castillo et al., 2022). Finally, our study found a significant decrease in the severity and frequency of IBS symptoms following ISTDP. These findings are in line with prior research suggesting that short-term psychodynamic psychotherapy is effective in the treatment of MUS (Lilliengren, 2017; Abbass et al., 2021). More specifically, in a review of 11 randomized controlled trials, two control trials, and ten case series studies, Russell et al. (2022) found that the ISTDP hold promises as a treatment modality for MUS. Psychodynamic treatments such as ISTDP propose that somatic symptoms are primarily caused by implicit emotional processing and distorted bodily awareness. According to ISTDP, these symptoms are manifestations of a patient’s automatic, unconscious, and habitual avoidance of emotions, leading to physiological states in the body (Russell et al., 2016, 2017). ISTDP aims to enhance patients’ self-understanding, including recognizing triggers, warning signs, and emotions associated with their symptoms (Town et al., 2019; Malda Castillo et al., 2022). By fostering the articulation and recognition of emotions, ISTDP empowers individuals to gain a deeper understanding of their life experiences. Moreover, the constructive impact of ISTDP on ER and defense mechanisms which was demonstrated in this study may elucidate why the treatment group experienced a notable reduction in both the severity and frequency of their IBS symptoms.

The findings of this study should be interpreted in light of several limitations. Firstly, the study did not differentiate between subtypes of IBS. Future research could benefit from addressing this aspect to provide a more nuanced understanding of IBS management. Secondly, the employment of self-report measures to evaluate outcome variables introduces the potential for biased data due to inaccuracies, social desirability bias, and shared method variance. Finally, the study’s sample size was constrained, emphasizing the need for future research with larger and more diverse samples to improve the generalizability and robustness of the findings.

## Conclusion

This RCT furnishes compelling empirical support for the effectiveness of ISTDP in treating individuals with IBS. The results demonstrated the substantial promise of ISTDP as a pivotal intervention in alleviating IBS symptoms. Meanwhile, rigorous inquiry into the change mechanisms of the ISTDP specifically with a qualitative approach is imperative for refining the clinical application of ISTDP, ensuring its optimal integration within the broader framework of IBS care.

## Data availability statement

The raw data supporting the conclusions of this article will be made available by the authors, without undue reservation.

## Ethics statement

The studies involving humans were approved by Research Deputy of Iran University of Medical Sciences. The studies were conducted in accordance with the local legislation and institutional requirements. The participants provided their written informed consent to participate in this study.

## Author contributions

FS: Conceptualization, Data curation, Formal analysis, Investigation, Methodology, Project administration, Software, Writing – original draft, Writing – review & editing. MD: Conceptualization, Project administration, Supervision, Writing – review & editing. FL: Conceptualization, Project administration, Supervision, Writing – review & editing. MeM: Project administration, Supervision, Writing – review & editing. MaM: Project administration, Supervision, Writing – review & editing.

## References

[ref1] Abbass A. Lumley M. A. Town J. Holmes H. Luyten P. Cooper A.. (2021). Short-term psychodynamic psychotherapy for functional somatic disorders: a systematic review and meta-analysis of within-treatment effects. J. Psychosom. Res. 145:110473. doi: 10.1016/j.jpsychores.2021.110473, PMID: 33814192

[ref2] Abbass A. Town J. Driessen E. (2012). Intensive short-term dynamic psychotherapy: a systematic review and Meta-analysis of outcome research. Harv. Rev. Psychiatry 20, 97–108. doi: 10.3109/10673229.2012.677347, PMID: 22512743

[ref3] American Psychiatric Association. (2013). Diagnostic and statistical manual of mental disorders (5th). New York: American Psychiatric Pub.

[ref4] Andrews G. Singh M. Bond M. (1993). The defense style questionnaire. J. Nerv. Ment. Dis. 181, 246–256. doi: 10.1097/00005053-199304000-00006 8473876

[ref5] Begley T. M. (1994). Expressed and suppressed anger as predictors of health complaints. J. Organ. Behav. 15, 503–516. doi: 10.1002/job.4030150603

[ref6] Besharat M. A. Bazzazian S. (2013). Psychometri properties of the cognitive emotion regulation questionnaire in a sample of Iranian population. Nurs. Midwif. J. 24, 61–70.

[ref7] Bokic T. Storr M. Schicho R. (2015). Potential causes and present pharmacotherapy of irritable bowel syndrome: an overview. Pharmacology 96, 76–85. doi: 10.1159/000435816, PMID: 26139425 PMC4541721

[ref8] Brandão T. Tavares R. Schulz M. S. Matos P. M. (2016). Measuring emotion regulation and emotional expression in breast cancer patients: a systematic review. Clin. Psychol. Rev. 43, 114–127. doi: 10.1016/j.cpr.2015.10.002, PMID: 26520599

[ref9] Chang L. Lacy B. E. Spiegel B. M. (2010). An evidence-based approach to therapy in IBS-D: a case study compendium. Gastroenterol. Hepatol. 6, 1–12. PMID: 22570639 PMC3338169

[ref10] Chey W. D. Kurlander J. Eswaran S. (2015). Irritable bowel syndrome: a clinical review. JAMA 313, 949–958. doi: 10.1001/jama.2015.0954 25734736

[ref11] Cooper A. Abbass A. Town J. (2017). Implementing a psychotherapy Service for Medically Unexplained Symptoms in a primary care setting. J. Clin. Med. 6:109. doi: 10.3390/jcm6120109, PMID: 29186054 PMC5742798

[ref12] Craske M. G. Wolitzky-Taylor K. B. Labus J. Wu S. Frese M. Mayer E. A.. (2011). A cognitive-behavioral treatment for irritable bowel syndrome using interoceptive exposure to visceral sensations. Behav. Res. Ther. 49, 413–421. doi: 10.1016/j.brat.2011.04.001, PMID: 21565328 PMC3100429

[ref13] Cretton A. Brown R. J. Curt LaFrance W. J. Aybek S. (2020). What does neuroscience tell us about the conversion model of functional neurological disorders? J. Neuropsychiatry Clin. Neurosci. 32, 24–32. doi: 10.1176/appi.neuropsych.19040089, PMID: 31619119

[ref14] Eijsbouts C. Zheng T. Kennedy N. A. Bonfiglio F. Anderson C. A. Moutsianas L.. (2021). Genome-wide analysis of 53,400 people with irritable bowel syndrome highlights shared genetic pathways with mood and anxiety disorders. Nat. Genet. 53, 1543–1552. doi: 10.1038/s41588-021-00950-8 34741163 PMC8571093

[ref15] Farzadkia M. Farhangi A. Abolghasemi S. (2023). Effectiveness of mindfulness-based stress reduction and intensive short-term dynamic psychotherapy in improving mental health and mitigating alexithymia in fibromyalgia patients. Int. J. Musculosk. Pain Prevent. 8, 836–845.

[ref16] First M. B. Gibbon M. (2004). “The structured clinical interview for DSM-IV Axis I disorders (SCID-I) and the structured clinical interview for DSM-IV Axis II disorders (SCID-II)” in Comprehensive handbook of psychological assessment, Vol. 2: Personality assessment. eds. Hilsenroth M. J. Segal D. L. (Hoboken: John Wiley & Sons, Inc)

[ref17] Frederickson J. (2013). Co-creating change: effective dynamic therapy techniques. California: Seven Leaves Press.

[ref18] Frederickson J. J. Messina I. Grecucci A. (2018). Dysregulated anxiety and dysregulating defenses: toward an emotion regulation informed dynamic psychotherapy [perspective]. Front. Psychol. 9:2054. doi: 10.3389/fpsyg.2018.02054, PMID: 30455650 PMC6230578

[ref19] Girden E. R. (1992). ANOVA: Repeated measures. London: Sage Publications, Inc.

[ref20] Gratz K. L. Roemer L. (2004). Multidimensional assessment of emotion regulation and dysregulation: development, factor structure, and initial validation of the difficulties in emotion regulation scale. J. Psychopathol. Behav. Assess. 26, 41–54. doi: 10.1023/B:JOBA.0000007455.08539.94

[ref21] Gross J. J. (2013). Handbook of emotion regulation. New York: Guilford publications.

[ref22] Gross J. J. Jazaieri H. (2014). Emotion, emotion regulation, and psychopathology: an affective science perspective. Clin. Psychol. Sci. 2, 387–401. doi: 10.1177/2167702614536164

[ref23] Gross J. J. Muñoz R. F. (1995). Emotion regulation and mental health. Clin. Psychol. Sci. Pract. 2, 151–164. doi: 10.1111/j.1468-2850.1995.tb00036.x

[ref24] Guan Q. (2019). A comprehensive review and update on the pathogenesis of inflammatory bowel disease. J Immunol Res 2019, 7247238–7247216. doi: 10.1155/2019/7247238, PMID: 31886308 PMC6914932

[ref25] Guthrie E. Creed F. Dawson D. Tomenson B. (1993). A randomised controlled trial of psychotherapy in patients with refractory irritable bowel syndrome. Br. J. Psychiatry 163, 315–321. doi: 10.1192/bjp.163.3.315 8401959

[ref26] Heidari Nasab L. Mansouri M. Azadfallah P. Shaieeri M. R. (2007). Validity and reliability of Defens style Questionaire (DSQ-40) in Iranian samples. Clin. Psychol. Pers. 5, 11–27.

[ref27] Hetterich L. Stengel A. (2020). Psychotherapeutic interventions in irritable bowel syndrome. Front. Psych. 11:286. doi: 10.3389/fpsyt.2020.00286, PMID: 32425821 PMC7205029

[ref28] Ihilevich D. Gleser G. C. (1986). Defense mechanisms: their classification, correlates, and measurement with the defense mechanisms inventory. Lyon: DMI Associates.

[ref29] Jung H. K. Kim Y. H. Park J. Y. Jang B. H. Park S. Y. Nam M. H.. (2014). Estimating the burden of irritable bowel syndrome: analysis of a nationwide korean database. J. Neurogastroenterol. Motil. 20, 242–252. doi: 10.5056/jnm.2014.20.2.242, PMID: 24840377 PMC4015204

[ref30] Kano M. Hamaguchi T. Itoh M. Yanai K. Fukudo S. (2007). Correlation between alexithymia and hypersensitivity to visceral stimulation in human. Pain 132, 252–263. doi: 10.1016/j.pain.2007.01.032, PMID: 17360119

[ref31] Keough M. E. Timpano K. R. Zawilinski L. L. Schmidt N. B. (2011). The association between irritable bowel syndrome and the anxiety vulnerability factors:body vigilance and discomfort intolerance. J. Health Psychol. 16, 91–98. doi: 10.1177/1359105310367689 20631041

[ref32] Knowles S. R. Austin D. W. Sivanesan S. Tye-Din J. Leung C. Wilson J.. (2017). Relations between symptom severity, illness perceptions, visceral sensitivity, coping strategies and well-being in irritable bowel syndrome guided by the common sense model of illness. Psychol. Health Med. 22, 524–534. doi: 10.1080/13548506.2016.1168932 27045996

[ref33] Koechlin H. Coakley R. Schechter N. Werner C. Kossowsky J. (2018). The role of emotion regulation in chronic pain: a systematic literature review. J. Psychosom. Res. 107, 38–45. doi: 10.1016/j.jpsychores.2018.02.002, PMID: 29502762

[ref34] Kopczyńska M. Mokros Ł. Pietras T. Małecka-Panas E. (2018). Quality of life and depression in patients with irritable bowel syndrome. Prz. Gastroenterol. 13, 102–108. doi: 10.5114/pg.2018.75819, PMID: 30002768 PMC6040097

[ref35] Laird K. T. Tanner-Smith E. E. Russell A. C. Hollon S. D. Walker L. S. (2016). Short-term and long-term efficacy of psychological therapies for irritable bowel syndrome: a systematic review and Meta-analysis. Clin. Gastroenterol. Hepatol. 14, 937–947.e4. doi: 10.1016/j.cgh.2015.11.020, PMID: 26721342

[ref36] Laird K. T. Tanner-Smith E. E. Russell A. C. Hollon S. D. Walker L. S. (2017). Comparative efficacy of psychological therapies for improving mental health and daily functioning in irritable bowel syndrome: a systematic review and meta-analysis. Clin. Psychol. Rev. 51, 142–152. doi: 10.1016/j.cpr.2016.11.001, PMID: 27870997

[ref37] Lee C. Doo E. Choi J. M. Jang S. H. Ryu H. S. Lee J. Y.. (2017). The increased level of depression and anxiety in irritable bowel syndrome patients compared with healthy controls: systematic review and Meta-analysis. J. Neurogastroenterol. Motil. 23, 349–362. doi: 10.5056/jnm16220, PMID: 28672433 PMC5503284

[ref38] Lilliengren P. (2017). Comprehensive compilation of randomized controlled trials (RCTs) involving psychodynamic treatments and interventions.

[ref39] Loddo I. Romano C. (2015). Inflammatory bowel disease: genetics, epigenetics, and pathogenesis [Mini review]. Front. Immunol. 6:551. doi: 10.3389/fimmu.2015.00551, PMID: 26579126 PMC4629465

[ref40] Malda Castillo J. Beton E. Coman C. Howell B. Burness C. Martlew J.. (2022). Three sessions of intensive short-term dynamic psychotherapy (ISTDP) for patients with dissociative seizures: a pilot study. Psychoanal. Psychother. 36, 81–104. doi: 10.1080/02668734.2021.2018623

[ref41] Masaeli N. Kheirabadi G. R. Afshar H. Daghaghzadeh H. Maracy M. R. Assadolahi F.. (2013). Validity, reliability, and factor analysis of Persian version of quality of life questionnaire for irritable bowel syndrome (IBS-QOL-34). J. Res. Med. Sci. 18, 492–496. PMID: 24250698 PMC3818619

[ref42] Mazaheri M. (2015). Difficulties in emotion regulation and mindfulness in psychological and somatic symptoms of functional gastrointestinal disorders. Iran J. Psych. Behav. Sci. 9:e954. doi: 10.17795/ijpbs-954, PMID: 26834811 PMC4733315

[ref43] Mehboodi K. Mohammadi N. Rahimi C. Sarafraz M. R. (2022). The efficacy of intensive short-term dynamic psychotherapy (ISTDP) on self-esteem, emotion regulation, and defense mechanisms in men with social anxiety disorder [اثربخشی روان‌درمانی پویشی فشرده کوتاه‌مدت بر حرمت خود، نظم‌جویی هیجان و مکانیسم‌های ‌دفاعی در مردان مبتلا به اختلال اضطراب اجتماعی]. Psychol. Sci. 21, 461–474. doi: 10.52547/JPS.21.111.461

[ref44] Pakdel H. Shorabi F. Haji Alizade K. (2022). The efficacy of intensive short-term dynamic psychotherapy on job stress coping strategies, health-related quality of life, and self-efficacy of Iran air traffic controler staff [applicable]. J. Psychol. Sci. 21, 543–558. doi: 10.52547/jps.21.111.543

[ref45] Patrick D. L. Drossman D. A. Frederick I. O. Dicesare J. Puder K. L. (1998). Quality of life in persons with irritable bowel syndrome (development and validation of a new measure). Dig. Dis. Sci. 43, 400–411. doi: 10.1023/A:1018831127942, PMID: 9512138

[ref46] Pellissier S. Bonaz B. (2017). “Chapter eleven - The place of stress and emotions in the irritable bowel syndrome” in Vitamins and hormones. ed. Litwack G. (New York: Academic Press) 10.1016/bs.vh.2016.09.005 28061975

[ref47] Phillips K. Wright B. J. Kent S. (2013). Psychosocial predictors of irritable bowel syndrome diagnosis and symptom severity. J. Psychosom. Res. 75, 467–474. doi: 10.1016/j.jpsychores.2013.08.002, PMID: 24182637

[ref48] Pokroy R. Mayer A. Stuart A. D. Pretorius H. G. (1999). Coping styles and defense mechanisms utilised by patients suffering from irritable bowel syndrome. Health SA Gesondheid 4:7. doi: 10.4102/hsag.v4i1.8

[ref49] Prat F. Malak N. A. Pelletier G. Buffet C. Fritsch J. Choury A. D.. (1996). Biliary symptoms and complications more than 8 years after endoscopic sphincterotomy for choledocholithiasis. Gastroenterology 110, 894–899. doi: 10.1053/gast.1996.v110.pm8608900, PMID: 8608900

[ref50] Russell L. Abbass A. Allder S. (2022). A review of the treatment of functional neurological disorder with intensive short-term dynamic psychotherapy. Epilepsy Behav. 130:108657. doi: 10.1016/j.yebeh.2022.108657, PMID: 35390566

[ref51] Russell L. A. Abbass A. A. Allder S. J. Kisely S. Pohlmann-Eden B. Town J. M. (2016). A pilot study of reduction in healthcare costs following the application of intensive short-term dynamic psychotherapy for psychogenic nonepileptic seizures. Epilepsy Behav. 63, 17–19. doi: 10.1016/j.yebeh.2016.07.017, PMID: 27541836

[ref52] Russell L. Turner A. Yates P. (2017). A preliminary evaluation of intensive short-term dynamic psychotherapy within a functional neurological symptoms service. Neuropsychologist 1, 25–32. doi: 10.53841/bpsneur.2017.1.4.25

[ref53] Saeed F. Salehi M. Alavi K. Ajdarkosh H. Kashaninasab F. Nasr Esfahani F. (2019). Defense mechanisms in patients with irritable bowel syndrome and their relationship with symptom severity and quality of life. Middle East J. Dig. Dis. 11, 158–165. doi: 10.15171/mejdd.2019.143, PMID: 31687115 PMC6819962

[ref54] Saha L. (2014). Irritable bowel syndrome: pathogenesis, diagnosis, treatment, and evidence-based medicine. World J. Gastroenterol. 20, 6759–6773. doi: 10.3748/wjg.v20.i22.6759, PMID: 24944467 PMC4051916

[ref55] Selvi K. Bozo Özen Ö. (2022). Group comparison of individuals with and without irritable bowel syndrome in terms of psychological and lifestyle-related factors. J. Psych. Neurol. Sci. 35, 13–23. doi: 10.14744/DAJPNS.2022.00167

[ref56] Shah K. Ramos-Garcia M. Bhavsar J. Lehrer P. (2020). Mind-body treatments of irritable bowel syndrome symptoms: an updated meta-analysis. Behav. Res. Ther. 128:103462. doi: 10.1016/j.brat.2019.103462, PMID: 32229334

[ref57] Sibelli A. Chalder T. Everitt H. Chilcot J. Moss-Morris R. (2018). Positive and negative affect mediate the bidirectional relationship between emotional processing and symptom severity and impact in irritable bowel syndrome. J. Psychosom. Res. 105, 1–13. doi: 10.1016/j.jpsychores.2017.11.016, PMID: 29332625

[ref58] Stanculete M. F. Căpățînă O. Pojoga C. Surdea-Blaga T. (2019). Anger mediates the relationship between pain and depression in irritable bowel syndrome [article]. J. Gastrointest. Liver Dis. 28, 415–419. doi: 10.15403/jgld-533, PMID: 31826066

[ref59] Town J. M. Lomax V. Abbass A. A. Hardy G. (2019). The role of emotion in psychotherapeutic change for medically unexplained symptoms. Psychother. Res. 29, 86–98. doi: 10.1080/10503307.2017.1300353, PMID: 28287345

[ref60] Trindade I. A. Melchior C. Törnblom H. Simrén M. (2022). Quality of life in irritable bowel syndrome: exploring mediating factors through structural equation modelling. J. Psychosom. Res. 159:110809. doi: 10.1016/j.jpsychores.2022.110809, PMID: 35649318

[ref61] Vafa Z. Azizi M. Elhami Athar M. (2021). Predicting academic alienation from emotion dysregulation, social competence, and peer relationships in school-attending girls: a multiple-regression approach. Front. Psychol. 12:755952. doi: 10.3389/fpsyg.2021.755952, PMID: 35035367 PMC8759297

[ref62] van Middendorp H. Geenen R. Sorbi M. J. Hox J. J. Vingerhoets A. J. J. M. van Doornen L. J. P.. (2005). Styles of emotion regulation and their associations with perceived health in patients with rheumatoid arthritis. Ann. Behav. Med. 30, 44–53. doi: 10.1207/s15324796abm3001_6, PMID: 16097905

[ref63] Weibert E. Stengel A. (2019). The role of psychotherapy in the treatment of irritable bowel syndrome. Psychother. Psychosom. Med. Psychol. 69, 360–371. doi: 10.1055/a-0829-6990 (Die Rolle Der Psychotherapie Beim Reizdarmsyndrom.), PMID: 30731513

[ref64] Yıldız M. A. Duy B. (2019). The predictive role of emotion regulation strategies on depressive and psychosomatic symptoms in adolescents. Curr. Psychol. 38, 387–396. doi: 10.1007/s12144-017-9616-6

[ref65] Zomorrodi S. Rasoulzadeh Tabatabaie S. K. Azadfallah P. Ebrahimidaryani N. Arbabi M. (2015). Long term effects of mindfulness on quality of life in irritable bowel syndrome. Iran. J. Psychiatry 10, 100–105. PMID: 26884786 PMC4752522

